# Hemophagocytic lymphohistiocytosis induced by radio-chemo-immunotherapy: a case report

**DOI:** 10.1007/s00066-025-02478-w

**Published:** 2025-10-14

**Authors:** Bryan Salazar-Zuniga, Lorenz Thurner, Tobias Mohr, Philipp Staber, Markus Hecht, Octavian Fleser

**Affiliations:** 1 Department of Radiotherapy and Radiation Oncology, Saarland University Medical Centre, Homburg, Germany; 2 Department of Hemato-Oncology, Saarland University Medical Centre, Homburg, Germany

**Keywords:** Hemophagocytic lymphohistiocytosis, Pembrolizumab, Cytokine storm, Immunotherapy adverse events, Radio-chemo-immunotherapy

## Abstract

**Background:**

Hemophagocytic lymphohistiocytosis (HLH) is a life-threatening hyperinflammatory syndrome characterized by supramaximal immune activation. Although rare, HLH has been increasingly recognized as an immune-related adverse event in patients undergoing immune checkpoint inhibitor (ICI) therapy.

**Case presentation:**

We report the case of a female patient treated with concomitant radio-chemo-immunotherapy for cervical cancer (according to the KEYNOTE-A18 trial). She developed HLH following a single dose of pembrolizumab, presenting initially with immune-mediated pneumonitis and subsequently with fever, prolonged pancytopenia, and elevated inflammatory markers. After intensive diagnostics, broad-spectrum anti-infective treatment and granulocyte colony-stimulating factor (G-CSF) stimulation was started, without improvement. The diagnosis was finally made by HLH-2004 criteria, strongly indicated by an H‑score of 251 (> 99% probability of HLH). The HLH was successfully treated with corticosteroids alone.

**Conclusion:**

This case highlights the importance of early recognition and aggressive management of HLH secondary to immunotherapy, particularly in patients presenting with unexplained fever, G‑CSF-refractory cytopenia, and hyperferritinemia.

## Introduction

Hemophagocytic lymphohistiocytosis (HLH) is a severe hyperinflammatory syndrome. The primary autosomal recessive form arises from mutations affecting genes involved in immune regulation and lymphocyte function. Immunopathologically, HLH is characterized by impaired cytotoxicity of NK cells and CD8⁺ T lymphocytes, leading to sustained activation of antigen-presenting cells. This dysfunction results in uncontrolled secretion of proinflammatory cytokines, including IFN‑γ, IL‑1, IL‑6, IL-18, and TNF‑α, which drive a cytokine storm. Activated macrophages further contribute by engulfing hematopoietic cells, producing cytopenia, hepatosplenomegaly, and a hyperinflammatory state that often mimics severe sepsis [1, 2]. Secondary or acquired HLH can be classified into five subtypes: infection-associated, malignancy-associated, autoimmune/inflammatory disease-related, drug-induced, and other-cause HLH [3, 4].

Hemophagocytic lymphohistiocytosis is increasingly recognized in adults and can affect individuals of all ages. Patients typically present with persistent fever with no evidence of infectious pathogens, cytopenia, elevated acute-phase reactants, and signs of sepsis [5, 6]. The diagnosis and management of immunotherapy-induced secondary HLH remain poorly defined, potentially resulting in diagnostic delays and inadequate countermeasures with progression to irreversible multiorgan failure and ultimately fatal outcomes [7].

The diagnosis of HLH in adults should be based on the HLH-2004 diagnostic criteria in conjunction with clinical assessment and patient history, including fever, splenomegaly, cytopenia affecting ≥ 2 lineages, hypertriglyceridemia and/or hypofibrinogenemia, hemophagocytosis, elevated ferritin, decreased NK cell activity, and increased soluble CD25 levels [1, 7]. Additional diagnostic tools, such as the H‑score (Table 1), can provide valuable aid in supporting the diagnosis in adults [8]. Immunotherapy-induced secondary HLH has rarely been reported, and due to the lack of clear diagnostic and therapeutic guidelines, we here present a case of immunotherapy-induced HLH successfully treated with corticosteroids.

**Table 1:** Simplified H‑score for the diagnosis of hemophagocytic lymphohistiocytosis.

Parameter	Criteria	Points
Temperature	< 38.4 °C	0
	38.4–39.4 °C	33
	> 39.4 °C	49
Cytopenia	1 lineage	0
	2 lineages	24
	3 lineages	34
Ferritin (ng/mL)	< 2000	0
	2000–6000	35
	> 6000	50
Triglycerides (mmol/L)	< 1.5	0
	1.5–4	44
	> 4	64
Fibrinogen (g/L)	> 2.5	0
	≤ 2.5	30
AST (U/L)	< 30	0
	≥ 30	19
Organomegaly	None	0
	Hepato- or Splenomegaly	23
	Both	38
Hemophagocytosis (BM aspirate)	Absent	0
	Present	35

The table summarizes the principal clinical and laboratory parameters used in the H‑score. The cumulative score corresponds to the estimated probability of hemophagocytic lymphohistiocytosis: ≤ 90 points (very low), 90–169 (low), 170–199 (moderate), ≥ 200 (high), and ≥ 250 (very high)

*AST* aspartate transaminase, *BM* bone marrow

## Case presentation

**Fig. 1:**
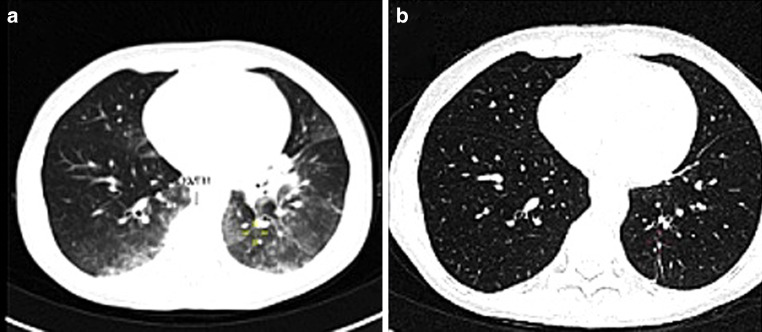
**a** Thoracic CT on January 23, 2025, showing immunotherapy-induced pneumonitis. **b** Follow-up CT on January 31, 2025, after corticosteroid therapy, showing resolution of pulmonary infiltrates

A 55-year-old female diagnosed with cervical cancer (FIGO IIIC) was undergoing concurrent chemoradiotherapy and immunotherapy, specifically with weekly cisplatin (40 mg/m^2^) and pembrolizumab (200 mg every 3 weeks). Following two doses of cisplatin and a single dose of pembrolizumab, the patient was admitted with dyspnea and fever. Infectious etiologies were excluded through comprehensive clinical assessment, laboratory analyses, radiological imaging, and repeated extensive microbiological testing. Thoracic CT revealed a diffuse atypical interstitial infiltrate, raising suspicion for immune-related pneumonitis. High-dose corticosteroid therapy was initiated, resulting in marked clinical and radiological improvement (Fig. 1). Corticosteroids were quickly tapered and discontinued.

Despite initial recovery 1 week later, the patient subsequently developed recurrent fever, pancytopenia, and elevated acute-phase reactants. Blood and urine cultures as well as tests for atypical pathogens and fungal infections returned negative. Additionally, two separate bronchoscopies showed no evidence of an infectious etiology. Under broad-spectrum antibiotics and hematopoietic growth factor support, the patient remained febrile, with worsening leukopenia (leukocyte count: 0.1 × 10^9^/L) and persistently elevated inflammatory markers (CRP 220 mg/l and ferritin 4679 ng/ml). An immune-related adverse event was hypothesized.

**Fig. 2:**
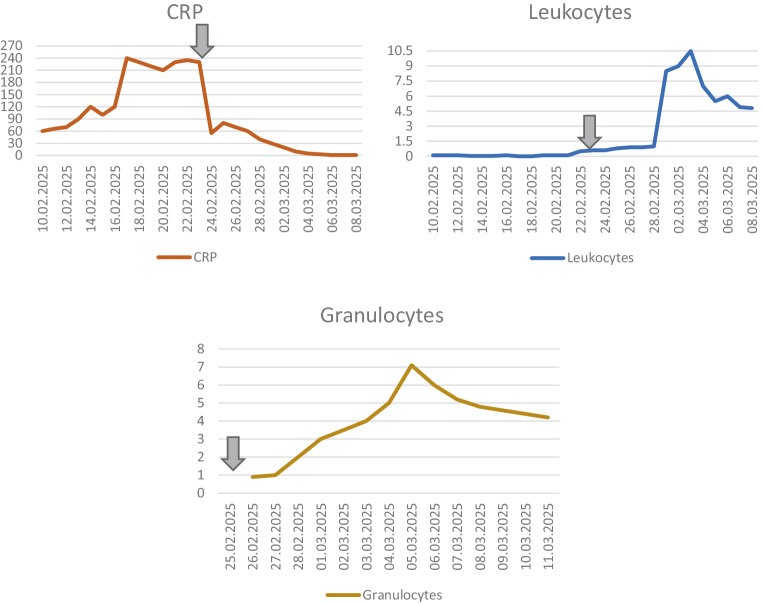
Evolution of laboratory values following initiation of corticosteroid therapy (arrow). C‑reactive protein (*CRP*) level decreased progressively, while leukocyte and granulocyte counts recovered over time

**Fig. 3:**
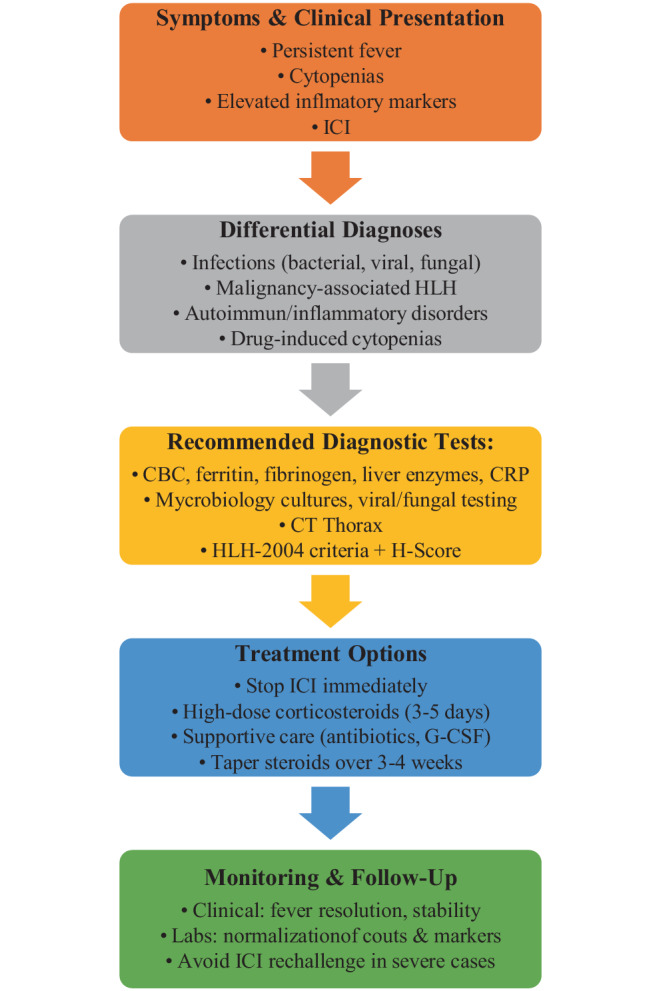
Visual algorithm supporting clinical decision-making in immunotherapy-induced hemophagocytic lymphohistiocytosis (*HLH*)

The patient was placed under reverse isolation to minimize the risk of secondary infections. During hospitalization, persistent fever was the only abnormal vital sign, with hemodynamic stability maintained at all times. Further diagnostic evaluation according to the HLH-2004 criteria revealed fever, cytopenia affecting three lineages, hyperferritinemia, hypofibrinogenemia, and elevated transaminases, strongly suggestive of HLH. The H‑score was calculated at 251, corresponding to > 99% probability of HLH. Based on these results, a diagnosis of pembrolizumab-induced HLH was hypothesized. Treatment with high-dose intravenous prednisolone 2 mg/kg bodyweight (100 mg/day) was initiated, leading to defervescence within 3 days, normalization of leukocyte counts, and gradual improvement of inflammatory markers (Fig. 2). Broad-spectrum antibiotics and G‑CSF were discontinued once infectious causes were definitively excluded. Corticosteroids were tapered and stopped after 3 weeks without relapse. A visual algorithm to support clinical decision-making is provided (Fig. 3).

At the time of last follow-up, the patient remained clinically stable with complete hematologic recovery, normal inflammatory markers, and no recurrence of HLH. Immunotherapy was not reintroduced due to the severity of the immune-related adverse event. Written informed consent for publication was obtained from the patient.

## Discussion

### Secondary HLH in adults

The pathophysiology of secondary HLH is heterogeneous and depends on the triggering factor. As a diagnostic approach, HLH can be categorized into malignancy-associated and non-malignancy-associated subgroups [9]. Due to the rarity and often delayed diagnosis of malignancy-associated HLH, fewer than 50% of patients receive appropriate treatment in a timely fashion [10].

Among oncologic patients, HLH can be further subdivided into three groups: malignancy-associated HLH, HLH during chemotherapy, and HLH-like cytokine storm induced by novel immunotherapies. Malignancy- and chemotherapy-related HLH are most frequently observed in patients with hematologic malignancies and are often triggered by viral infections [7, 11, 12]. The increasing use of immune checkpoint inhibitors has led to reports of cytokine release syndrome (CRS) associated with these therapies. Patients have been treated with CTLA‑4 and PD-1/PD-L1 checkpoint inhibitors [3, 13–16]. Early recognition of this syndrome, cessation of immunotherapy, and prompt corticosteroid treatment often result in rapid and complete resolution. [7, 9].

Our patient developed early, uncommon adverse effects after administration of pembrolizumab, with initial improvement under corticosteroids. Upon readmission, persistent fever, cytopenia, and elevated inflammatory markers without evidence of infection prompted consideration of alternative diagnoses.

The key to diagnosing HLH lies in maintaining a high index of suspicion in patients presenting with unexpected hyperinflammatory syndromes, especially those with persistent fever, hyperferritinemia, and pancytopenia [7, 9]. The HLH-2004 criteria, although developed for pediatric populations, remain a useful tool in adults as well. Additionally, the H‑score (Table 1) provides a strong aid to quantifying the probability of an HLH diagnosis. Our patient met both criteria, with an H‑score of 251 (99% probability). Genetic testing is not routinely recommended in adults presenting with HLH due to low yield [7].

### HLH-like cytokine storm induced by novel immunotherapies

This syndrome has been most frequently reported in patients with hematologic malignancies undergoing CAR-T cell therapy [17]. With the expanding use of immune checkpoint inhibitors (ICIs) in various solid tumors—especially melanoma—there has been an increasing number of secondary HLH cases attributed to immunotherapy. [3, 7, 14, 15, 19].

Immune checkpoints maintain immune homeostasis by attenuating T cell responses. Their inhibition with ICIs eliminates this regulatory control, resulting in excessive T cell recruitment and hyperactivation, potentially causing HLH-like toxicities. This phenomenon has been observed with anti-CTLA‑4, anti-PD‑1, and anti-PD-L1 therapies, whether used as monotherapy or in combination [14–16].

A query of the WHO pharmacovigilance database identified 38 cases of HLH, showing low co-occurrence with other immune-related adverse events or infections. The median onset was 6.7 weeks after initiating ICIs, and in 7 cases, HLH was the primary or contributing cause of death [18].

### Treatment

The HLH-94 protocol can serve as a reference template, with appropriate dose and frequency adjustments based on the patient’s condition. Recommended therapies include corticosteroids, etoposide, cyclosporine, and methotrexate, aimed at controlling the hyperinflammatory response by killing immune cells, thereby terminating the pathologic immune response [2, 3, 7].

In suspected cases of immunotherapy-induced HLH, discontinuation of ICIs and administration of high-dose corticosteroids for 3–5 days may suffice. In our case, corticosteroid therapy was initiated with 100 mg of prednisolone i.v. per day, resulting in marked clinical improvement within 3 days [7, 19]. Leukocyte and neutrophil counts recovered, and C‑reactive protein levels gradually returned to normal (Fig. 2), allowing for discontinuation of antibiotics and supportive therapies. The corticosteroid treatment was gradually and completely discontinued over a period of 3 weeks.

Rechallenging oncologic patients after severe grade 3 or 4 immune-related adverse events (irAEs) after ICI administration harbors an increased risk for relapse of irAEs [20]. In concordance with the ESMO Clinical Practice Guideline for diagnosis, treatment, and follow-up of toxicities from immunotherapy [21], in this case, we did not rechallenge the patient, especially considering that several studies and reviews have shown that the occurrence of irAEs in patients treated with checkpoint inhibitors, such as pembrolizumab, may be associated with a better clinical response and longer overall survival [22].

## Conclusion

Hemophagocytic lymphohistiocytosis (HLH) induced by immune checkpoint inhibitors is an increasingly recognized but potentially fatal complication. Clinicians should maintain a high index of suspicion for HLH in patients presenting with fever, hyperferritinemia, and cytopenia following immunotherapy. Early corticosteroid therapy may lead to full recovery if initiated promptly.
